# Lateral para-olecranon approach: surgical guide and anatomical considerations to the anconeus branch: is there a nerve-free zone?

**DOI:** 10.1007/s00068-022-02141-4

**Published:** 2022-10-20

**Authors:** Michael Plecko, Ulrike M. Schwarz, Gloria M. Hohenberger, Niels Hammer, Angelika M. Schwarz

**Affiliations:** 1 AUVA – Trauma Hospital (UKH) Styria | Graz, Teaching Hospital of the Medical University of Graz, Graz, Austria; 2 Division of Macroscopic and Clinical Anatomy, Gottfried Schatz Research Center, Medical University of Graz, Graz, Austria; 3 Department of Trauma Surgery, State Hospital Feldbach-Fürstenfeld, Feldbach, Austria; 4 Division of Medical Technology, Fraunhofer Institute for Machine Tools and Forming Technology (Fraunhofer IWU), Dresden, Germany; 5 Department of Trauma, Orthopaedics and Plastic Surgery, University Hospital of Leipzig, Leipzig, Germany; 6 Department of Orthopaedics and Trauma, Medical University of Graz, Graz, Austria

**Keywords:** Lateral para-olecranon approach, Anconeus branch, Triceps-on approach, Distal humeral fracture, Total elbow arthroplasty

## Abstract

**Purpose:**

In the last decades, total elbow arthroplasty, elbow osteosynthesis and revision surgery have been more popularized. The study aimed to assess the course of the anconeus branch of the radial nerve in relation to two variations of the lateral para-olecranon approach, considering iatrogenic nerve injuries.

**Methods:**

The study consisted of 120 upper extremities from 60 Thiel-embalmed human specimens. Two randomized versions of the lateral para-olecranon approach (centrally orientated: P1 and laterally orientated: P2) were performed. The olecranon and the intersection points to the anconeus branch of the radial nerve were determined as anatomical landmarks. The measurements were assessed by two independent observers. Differences were analyzed using the Student’s t test; associations were computed with the Pearson correlation (*r*). An alpha of 0.05 (*p*) and a confidence interval of 95% were set.

**Results:**

The intersection points averaged 12.3 cm (SD 1.8, range 8.2–16.8) for P1 versus 5.5 cm (SD 1.4, range 3.0–9.2) for P2 (*p* ≤ 0.001). Statistically significantly higher values for male and longer humeral specimens were revealed (all values: *p* < 0.05). Comparison of left and right sides yielded no difference. Excellent inter-rater agreements were found (ICC = 0.902, range 0.860–0.921). A correlation was evaluated between the humeral length and the distances in both approaches (P1: *r* = 0.550, *p* < 0.001, P2: *r* = 0.669, *p* < 0.001).

**Conclusion:**

The data presented here allow preservation of the anconeus branch. The P1 forms a potential advantage by owing a broader safe zone. Using the centrally orientated approach seems to provide adequate nerve protection during surgery for one of the motor branches for extension of the elbow joint and might result in improved postoperative benefits.

## Introduction

Osteosynthesis, total arthroplasty and revision surgery of the elbow region have become more commonly used treatments over the last few decades. In particular, the rate of fracture surgeries has increased due to the bimodal distribution and the demographic circumstances [1, 2]. Comminuted fractures of the distal humerus are considered challenging, as a result of the complexity of the regional anatomy and the multifragmentary pattern. The optimal treatment is surgery with locking plates and functional aftercare [2]. Total elbow arthroplasty is known as an alternative treatment option for specific fracture situations, whereby good results have been shown [1, 3–5].

The approach is perhaps the most crucial key point for a successful surgery in joint preserving and joint replacement strategies. In challenging cases, surgical exposure is usually gained through one of the various dorsal approaches. The technical differences mainly concern the mobilization of the triceps muscle. They can be broadly distinguished between (A) triceps-off approaches, where the muscle is detached from the olecranon, and (B) triceps-on approaches, where the major portion of the insertion is preserved [6–9].

The lateral para-olecranon approach (PA) is a triceps-on approach and has been gaining popularity due to the benefit of preserving the extensor mechanism. As reported by Studer et al. [6], it reflects the triceps of the posterior humerus on the medial side and splits the triceps muscle, so that the lateral aspect of the triceps tendon and the anconeus muscle is elevated off laterally as a single unit. The approach has been described for total elbow arthroplasty [6] and osteosynthesis of distal humeral fractures [7].

An anatomical structure at risk presented by this approach is the anconeus branch. The anconeus muscle innervation originates from the radial nerve and runs as anconeus branch through the medial head of the triceps to the anconeus muscle. This branch is part of the motor nerve supply for the extension in the elbow joint, as it innervates parts of the triceps muscle and the entire anconeus muscle [10].

The purpose of this study was to visualize the anconeus branch in the context of triceps-on surgery to provide a morphological basis for preserving the motor function of parts of the triceps muscle and the entire anconeus muscle while adequately visualizing the site of surgery. It was hypothesized that a defined triceps muscle-preserving technique allows for defining a broader safe zone for the anconeus branch.

## Materials and methods

### Study subjects

The postmortem sample included 120 paired upper extremities from 60 human adult specimens with a mean age of 79 years (SD 10.8 years; range 46–95 years, median 80 years). The average body mass index of all eligible body donors was 24 kg/m^2^ (SD 3.6 kg/m^2^; range 17.2–36.0 kg/m^2^, median 23.4 kg/m^2^). The study collective consisted of a balanced sex distribution—47% females (28/60) and 53% males (32/60)—whereby the upper extremities of both sides were evaluated.

All the specimens were in a Thiel-embalmed state, which provides tissues with similar consistency and flexibility compared to the fresh, unembalmed state [11–14]. The exclusion criteria involved obvious signs of trauma or severe deformities as well as visual evidence of prior surgeries or other pathologies of the musculoskeletal apparatus.

### Study design

In this in vitro study, two versions of the PA were characterized based on anatomical landmarks. The intersection point was defined inline by the approaches from specific anatomical landmarks to the anconeus branch. It was aimed to determine a safe zone for the nerve through these direct measurements. Absolute distances were used to minimize possible distortion when using correlations and for straightforwardness and clinical applicability.

The course of the anconeus branch was documented in all specimens, along with relevant adjacent anatomical structures. Its specific sensory branches were not evaluated. Additional research questions were defined as follows: How do (A) side, (B) sex and (C) humeral length affect the nerve relative to the approaches?

**Fig. 1:**
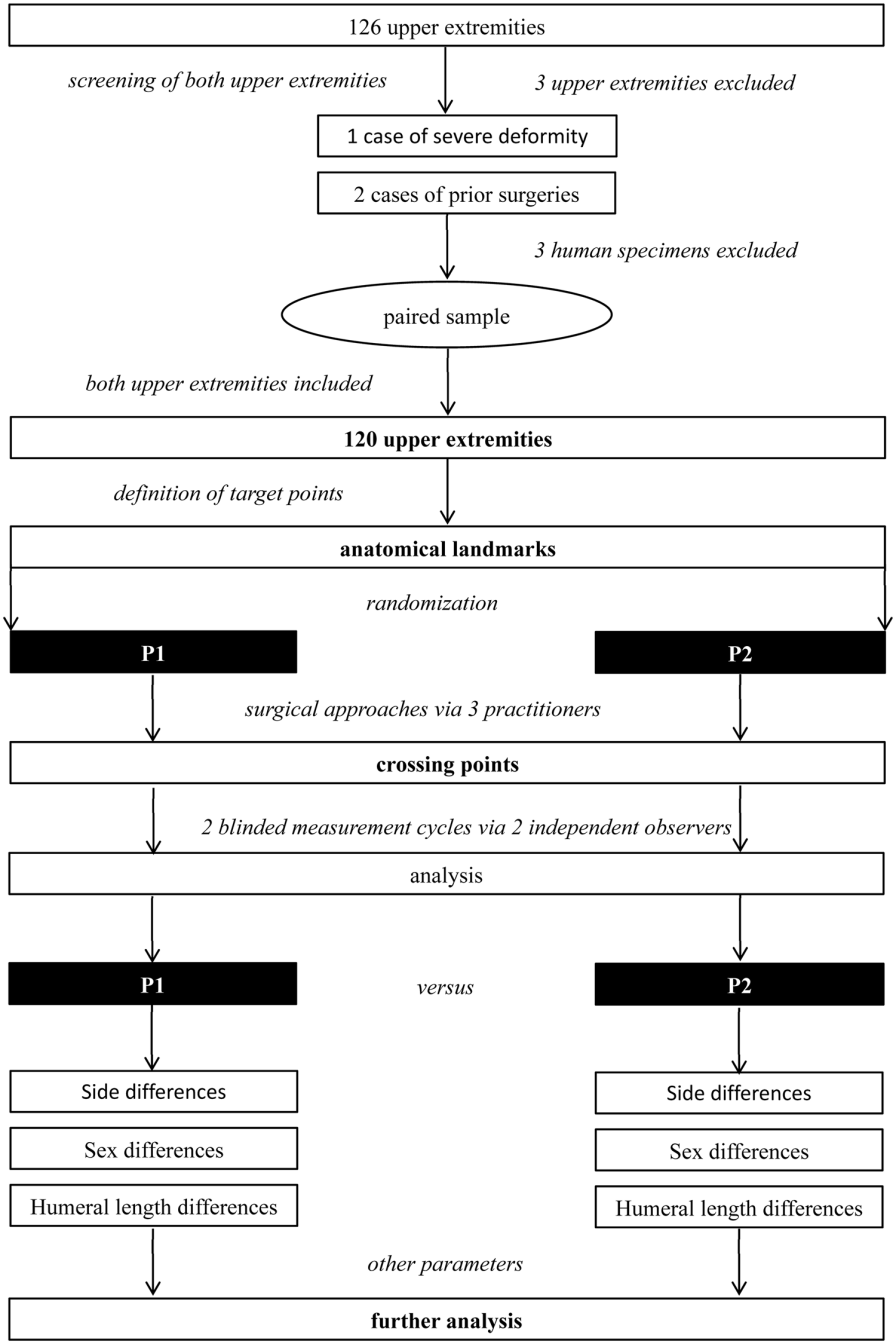
Flow chart of the study design. The measurement and research focuses are displayed. Primarily, 63 specimens were enclosed—60 of them were included for the approaches and measurements in the paired study sample. Worded differently, 120 upper extremities were analyzed. *P1* medially/centrally orientated lateral para-olecranon approach 1, *P2* laterally orientated lateral para-olecranon approach 2

The surgical approaches and dissections were carried out by a trauma surgeon (AS) and a student tutor (US) under observation by a trauma consultant (MP). The sequence was randomized regarding their performance to which approach would be tested first. All the measurements were investigated by two independent observers (AS and US) in separate sessions with an interval of 2 months without knowledge of each other’s ratings. The study design is illustrated in detail in Fig. 1.

### Testing methods and variable targets

#### Setup

**Fig. 2:**
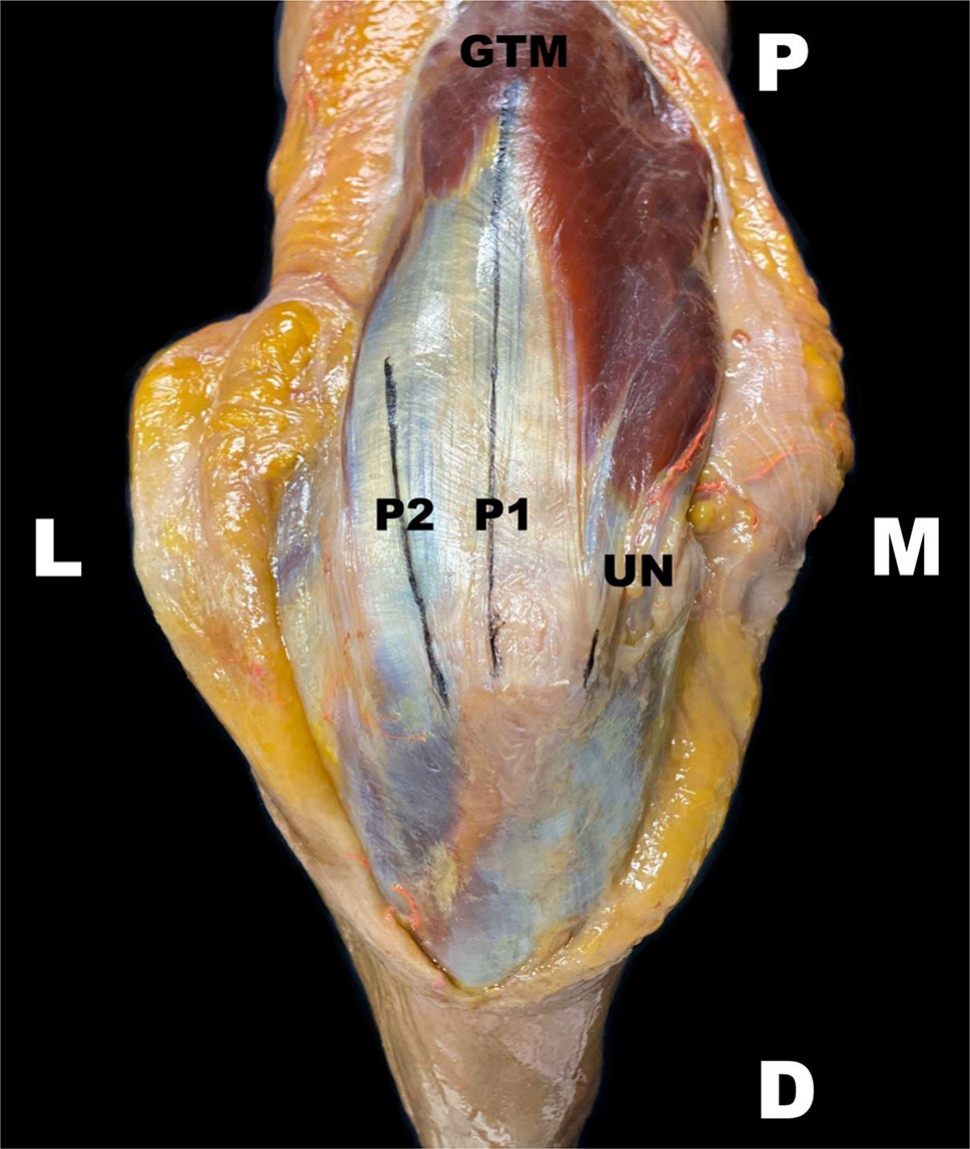
Scheme of approaches. The lines are marked in black to illustrate both surgical approaches. A left upper extremity is pictured in a dorsal view. *P1* medially/centrally orientated lateral para-olecranon approach 1, *P2* laterally orientated lateral para-olecranon approach 2, *D* distal, *M* medial, *O* medial border of the olecranon, *L* lateral, *P* proximal, *GTM* gap of the triceps muscle between the medial and lateral head, *UN* ulnar nerve

**Fig. 3:**
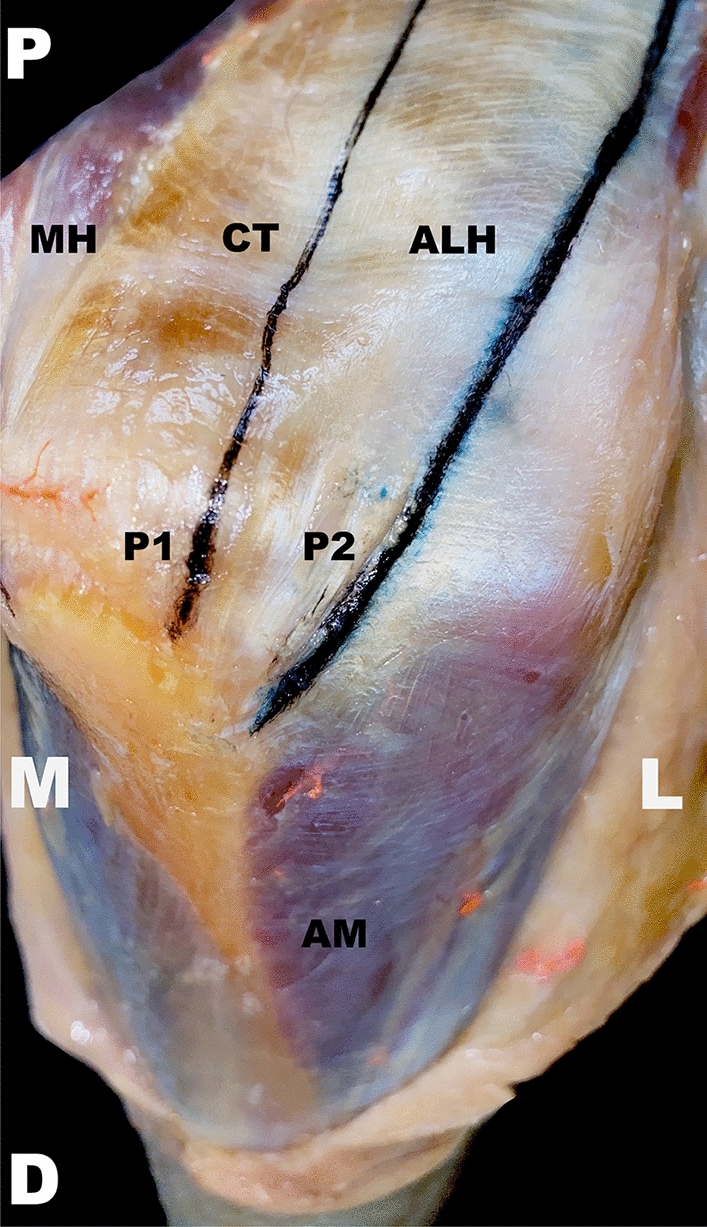
Approaches in a detailed aspect. The approaches are marked in black. The anconeus muscle can be viewed in its entirety. A right upper extremity is pictured in a dorsolateral view. *P1* medially/centrally orientated lateral para-olecranon approach 1, *P2* laterally orientated lateral para-olecranon approach 2, *AM* anconeus muscle, *ALH* aponeurosis of the lateral head, *CT* central tendon, *D* distal, *M* medial, *MH* medial head of the triceps muscle, *L* lateral, *P* proximal

All the specimens were placed in prone position, with the upper arm placed in approximately 90° abduction to the trunk. The elbow joint was fixed in 90° flexion, with the forearm in neutral position. The dorsal aspect of the entire upper arm and the proximal forearm was dissected by a subcutaneous medial and lateral flap, and the triceps muscle was visualized under the protection of its fascia. Afterward, the respective approach was marked with black pens (Figs. 2, 3).

#### Anatomical landmarks

**Fig. 4:**
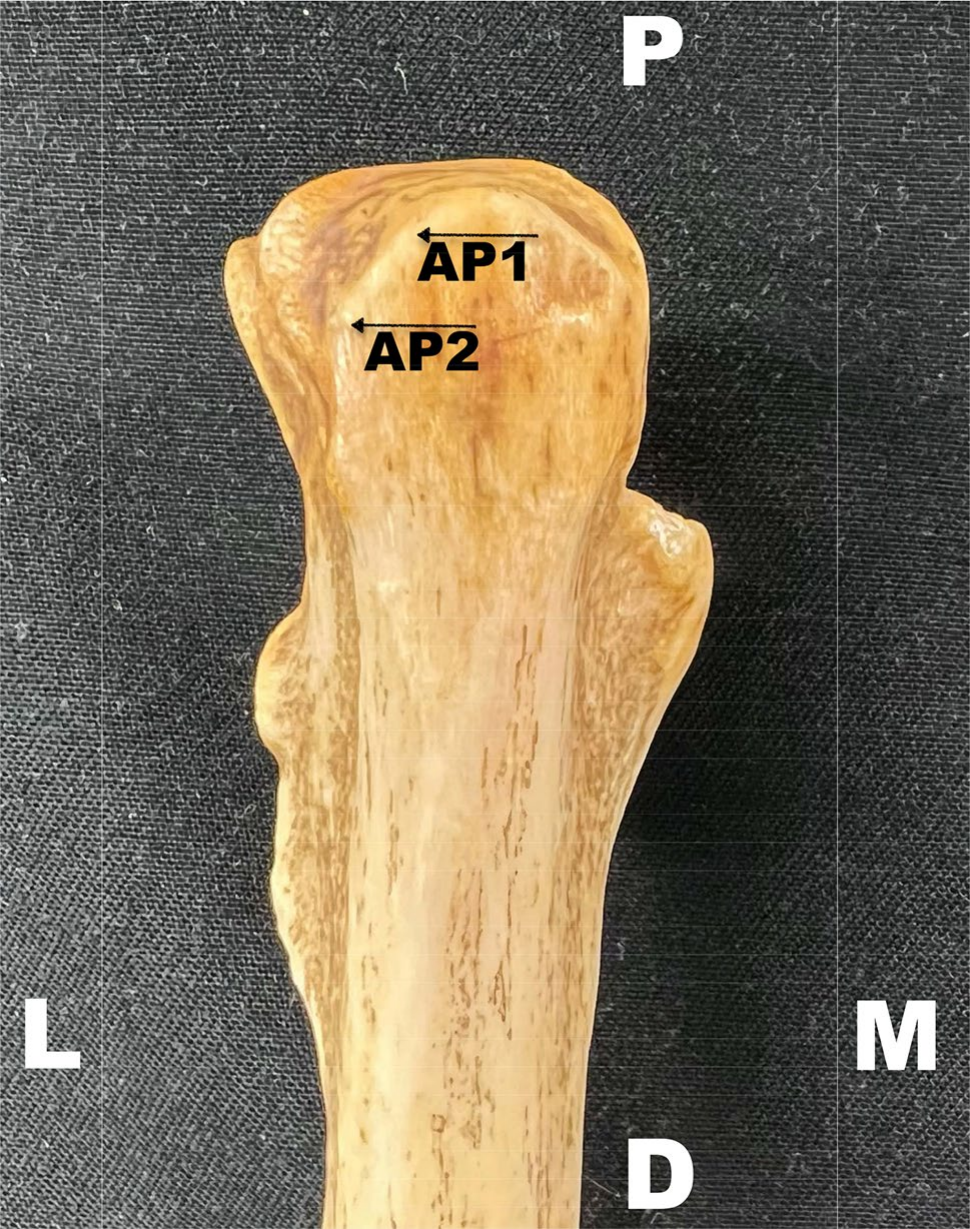
Bony anatomical landmarks. A proximal ulna is displayed with the two osseous reference points in detail. A left upper extremity is pictured in a dorsal view. *AP1* the lateral edge of the most proximal apex of the olecranon, *AP2* the most lateral edge of the entire olecranon, *D* distal, *M* medial, *MH* medial head of the triceps muscle, *L* lateral, *P* proximal

Easily reproducible structures were defined as anatomical landmarks. Two specific points on the olecranon defined the bony reference points (Fig. 4):

1. AP1: the lateral edge of the most proximal apex of the olecranon.
2. AP2: the most lateral edge of the entire olecranon.

#### Surgical technique and approaches

First, a Boyd approach [15] was carried out, whereas a small medial fascial strip was left. Next, a centrally orientated (P1) or laterally orientated (P2) approach or vice versa was performed sequentially. Both approaches were performed via longitudinal splitting of the triceps muscle in two different proximal directions as follows (Figs. 2, 3):

(A) P1: Access was gained in a line between AP1 and the gap between the medial and lateral head of the triceps muscle. This gap was located 2 cm distally from the insertion of the deltoid muscle, posteriorly directed toward the center of the humeral shaft. The muscle splitting was orientated parallel to the humeral shaft axis.
(B) P2: Access was gained in a line between AP2 parallel to the aponeurosis fiber direction of the lateral head of the triceps muscle.

### Measurement pattern

1. The humeral length was evaluated as the distance between the most proximal tip of the greater tubercle and the most distal aspect of the humeral capitulum. The radial nerve and its branches were exposed, and the anconeus branch was marked and its course documented. Care was taken to not manipulate the nerves’ original topography and course.
2. To assess the topographical aspect, one longitudinal and one transverse distance were defined starting from the olecranon. In the longitudinal orientation, the direct interval from the exit point of the anconeus branch at the radial nerve to the olecranon was examined. Here, the distal measure point was fixed at the midpoint of the olecranon tip. Afterward, to assess the transverse distance to the olecranon, the direct distance between the AP2 and the anconeus branch was evaluated in an orientation parallel to the transverse elbow joint axis.
3. As target point, the direct distances from AP1 and AP2 to the anconeus branch’s intersection points through each approach were measured.

**Fig. 5:**
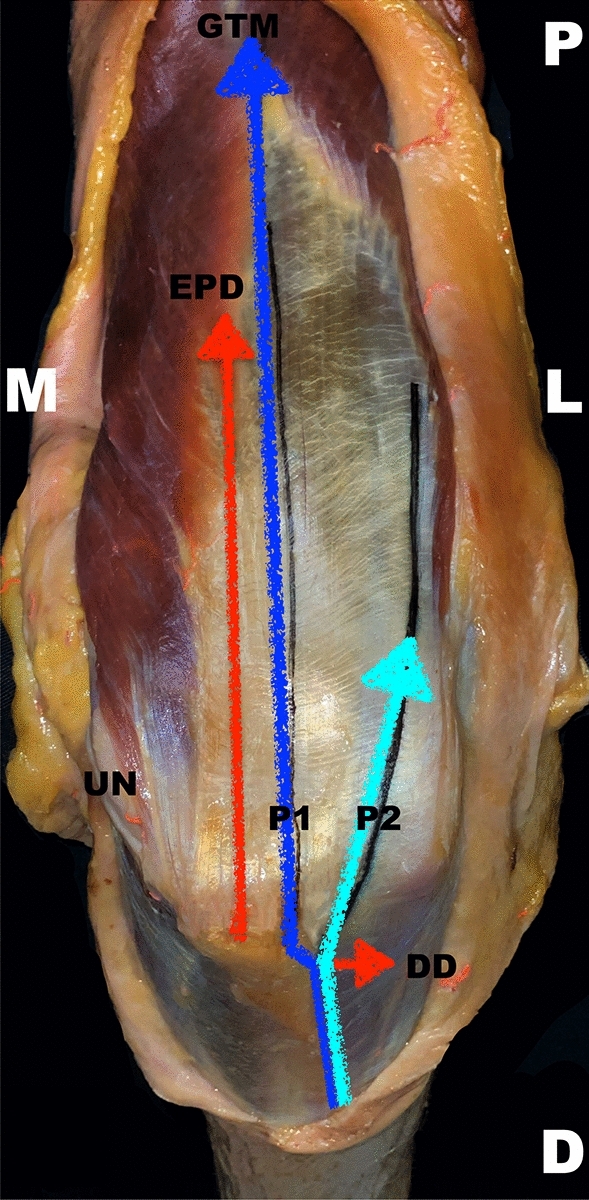
Schematic measurement directions. The measurement directions are highlighted through lines. Both approaches are marked via dark (P1) and light (P2) blue lines. A right upper extremity is pictured in a dorsal view. *P1* medially/centrally orientated lateral para-olecranon approach 1, *P2* laterally orientated lateral para-olecranon approach 2, *EPD* longitudinal distance from the middle of the apex of the olecranon to the exit point of the anconeus branch of the radial nerve, *D* distal, *DD* transverse direct distance from the most lateral edge of the olecranon to the anconeus branch, *GTM* gap of the triceps muscle between the medial and lateral head, *L* lateral, *M* medial, *P* proximal, *UN* ulnar nerve

**Fig. 6:**
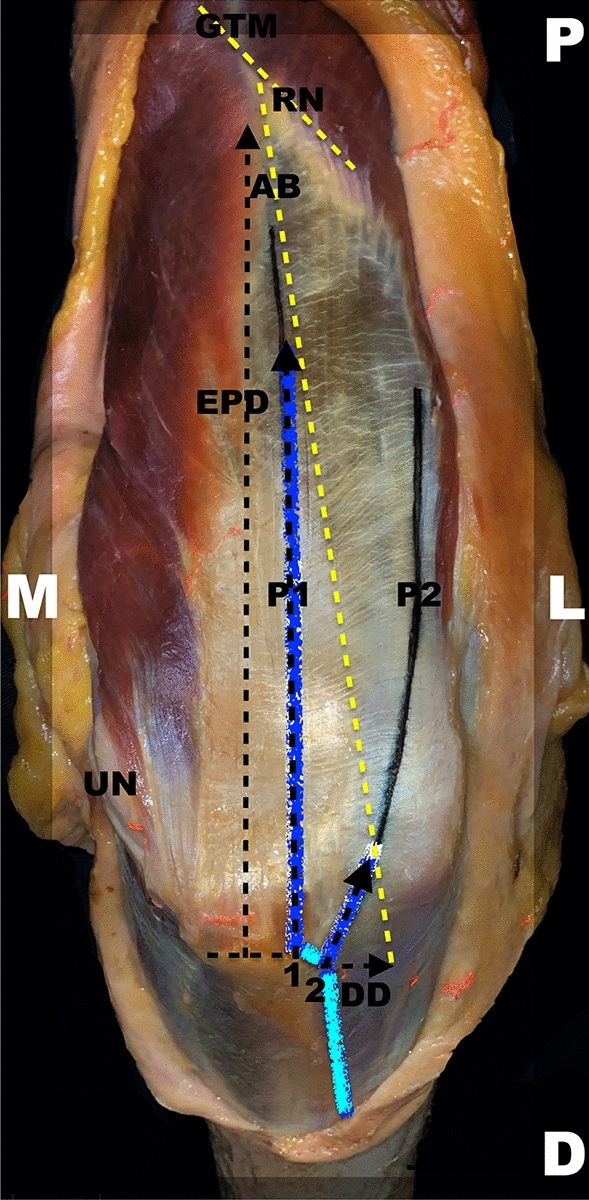
Schematic measurement pattern. All measured distances are pointed out with lines. Both main target points, meaning the direct distances of the intersection points of P1 as well as P2 with the anconeus branch, are underlined in blue. The topographical direct distances are shown as dashed lines in black from the bony anatomical landmarks to the nerve’s intersection points in a longitudinal and transverse direction. The nerves are displayed as dashed lines in yellow. A right upper extremity is pictured in a dorsal view. *P1* medially/centrally orientated lateral para-olecranon approach 1, *P2* laterally orientated lateral para-olecranon approach 2, *AB* anconeus branch, *EPD* longitudinal distance from the middle of the apex of the olecranon to the exit point of the anconeus branch of the radial nerve, *D* distal, *DD* transverse direct distance from the most lateral edge of the olecranon to the anconeus branch, *GTM* gap of the triceps muscle between the medial and lateral head, *L* lateral, *M* medial, *P* proximal, *RN* radial nerve, *UN* ulnar nerve

All the measurements were taken with a digital caliper rule with an accuracy of 0.01 mm (Emil Lux GmbH & Co. KG, Germany; Art. No. 572587) and quantified in centimeters. For a schematic depiction of measurement pattern, see Figs. 5 and 6.

### Statistical analysis

Statistical analyses were conducted using the SPSS software (IBM SPSS Statistics version 27, Armonk, NY, USA). Descriptive statistics were utilized for demographic variables. The continuous data were presented as mean, median, standard deviation (SD); and range via minimum and maximum. The variables satisfied the conditions of normal distribution.

Randomization for the sequences of P1 or P2 was done via the randomizer software [16]. The evaluation as to the sequence correlates with the extent of the safe zone was analyzed with the independent t test plus Levene’s test. Analyses of (a) side, (b) sex and (c) humeral length-specific differences were carried out with the independent *t* test plus Levene’s test. Inter-rater reliability was assessed with the intra-class type correlation coefficient ICC (two-way mixed, ICC 2.1) [17]. Linear regression analyses by the Pearson coefficients (*r*) were performed to look for potential (a) side, (b) sex and (c) humeral length-specific associations [18].

*p* values (*p*) with a significance level of *p* =  ≤ 0.05 were defined. Confidence intervals of 95% were computed. A post hoc power analysis was performed for the comparison of both used approaches (P1 and P2) with G*Power 3.1. [19]. According to an alpha of 0.05, it was shown that the sample size could achieve a power of 0.99 based on a two-tailed significance test [20].

## Results

### Anatomical guide

The humeral length averaged 30 cm (SD 2.3 cm; range 26.1–36.9 cm, median 29.6 cm). The transverse direct distance between the lateral tip of the olecranon and the anconeus branch averaged 1.1 cm (SD 0.3 cm; range 0.4–1.9 cm, median 1.0 cm). The mean interval between the tip of the olecranon and the exit point of the radial nerve was 17.7 cm (SD 1.0 cm; range 16.1–19.4 cm, median 17.6 cm).

**Fig. 7:**
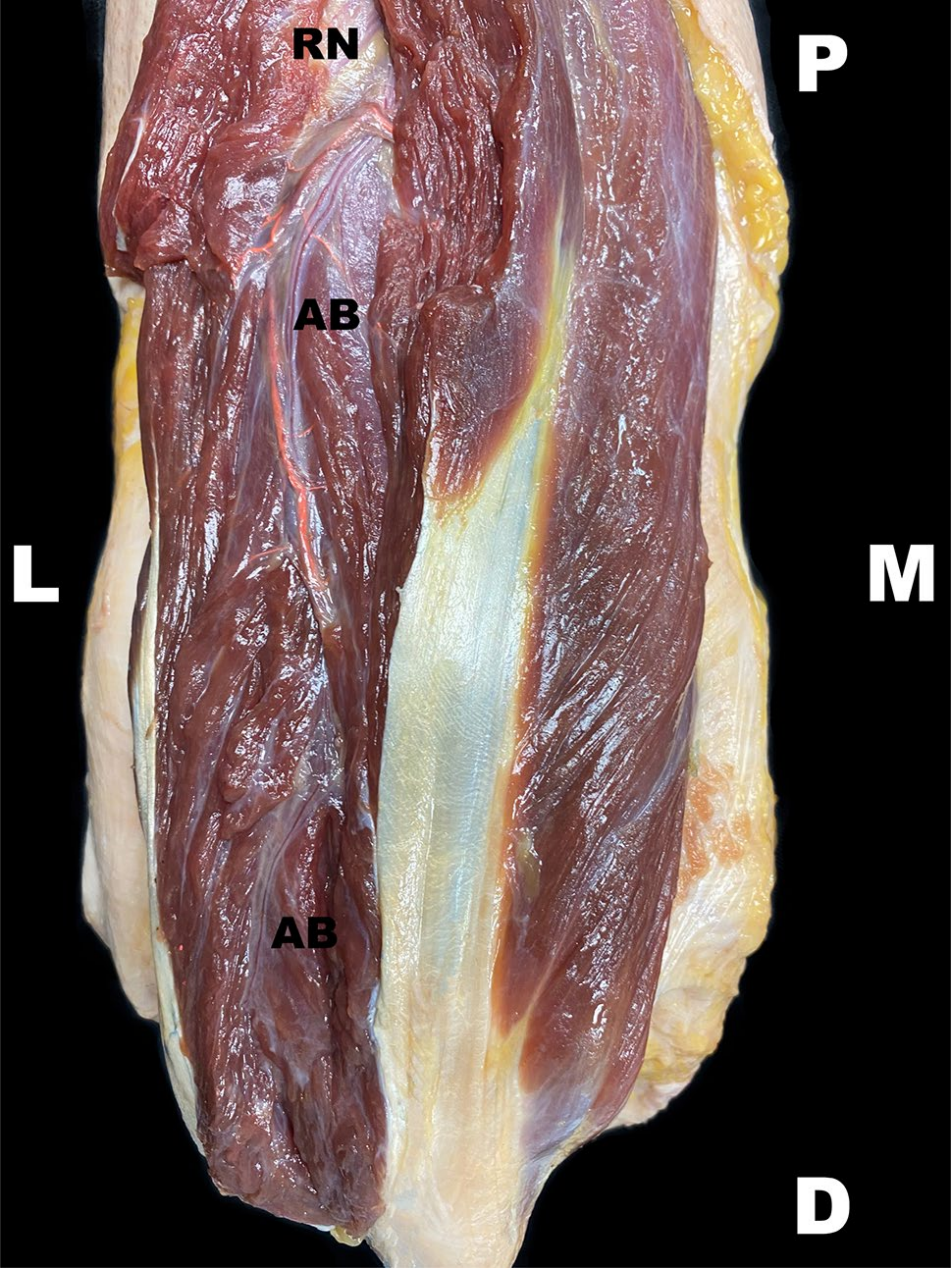
Course of the anconeus branch. The exit point of the radial nerve and its concomitant artery is put on view on the upper arm. The radial nerve is visualized in the proximal aspect of the picture. A left upper extremity is pictured in a dorsal view. *AB* anconeus branch, *RN* radial nerve, *D* distal, *M* medial, *O* medial border of the olecranon, *L* lateral, *P* proximal

**Fig. 8:**
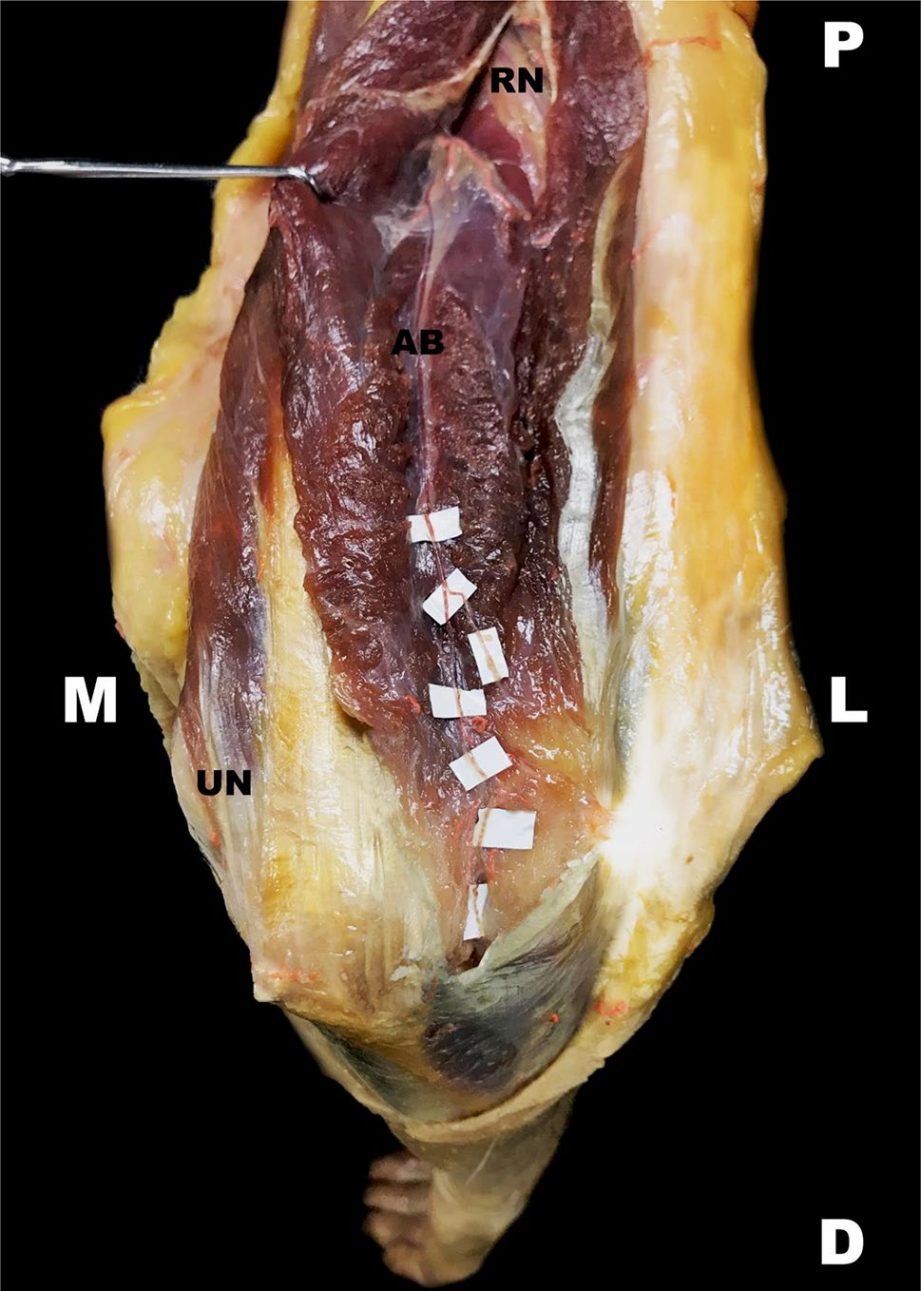
Full course of the anconeus branch. The anconeus branch and its course is pointed out. A close relationship to the elbow joint capsule as well as its transverse distance of about 1 cm from the lateral edge of the olecranon to the nerve can be viewed. A right upper extremity is pictured in a dorsal view. *AB* anconeus branch, *D* distal, *M* medial, *O* olecranon, *L* lateral, *P* proximal, *RN* radial nerve, *UN* ulnar nerve

The anconeus branch has been observed as the most distal direct branch of the radial nerve in the spiral groove. This branch was present in all specimens (100%, 120/120). A small-caliber artery accompanied the anconeus branch in all specimens along its course. The nerve ran through the medial head of the triceps muscle laterally and distally (Fig. 7). The anconeus branch exited the triceps muscle at the lower border of the triceps muscle proximal to the lateral epicondyle and reached the anconeus muscle laterally to the olecranon. Here, the nerve was located in proximity to the elbow joint capsule before entering the anconeus muscle (Fig. 8).

### Differences of both surgical approaches

The distances between the olecranon and the intersection point with the anconeus branch averaged 12.3 cm (SD 1.8 cm; range 8.2–16.8 cm, median 12.5 cm) for P1 and 5.5 cm (SD 1.4 cm; range 3.0–9.2 cm, median 5.5 cm) for P2, with the values being significantly different (*p* ≤ 0.001).

The correlation of humeral length and the anconeus branch’s intersection point showed an association in Pearson correlations for both approaches (P1: *r* = 0.550, *p* < 0.001, P2: *r* = 0.669, *p* < 0.001). No correlation was documented regarding the direct distance (*r* = 0.187, *p* < 0.285).

No significant difference was observed between the randomized sequences of P1 or P2 implementation. The mean distances were 12.4 cm (SD 1.8 cm; range 8.2–16.8 cm, median 12.5 cm) versus 12.1 cm (SD 1.6 cm; range 8.3–16.6 cm, median 12.2 cm) in P1 (*p* = 0.324). In P2, the mean distances were 5.5 cm (SD 1.2 cm; range 3.0–8.7 cm, median 5.6 cm) versus 5.6 cm (SD 1.3 cm; range 3.3–9.2 cm, median 5.7 cm) (*p* = 0.685). Furthermore, the inter-rater assessments resulted in excellent mean-rating agreements with an ICC of 0.902 (range 0.860–0.921), with no systematic difference between the 2 raters (*p* = 0.860).

### Side, sex and humeral length-depending differences

Assessing the variances by each approach, no significant difference was observed between sides (Table 1). A statistically significant difference was documented in males with longer distances in all parameters (Table 2). Similarly, statistically significantly increased values were observed in longer humeral bones (Table 3).

**Table 1:** Data analyzed per side

Parameter	Side	Mean (cm)	SD (cm)	Range (cm)	*p* value
P1	Right	12.3	1.8	8.2–16.5	0.710
	Left	12.2	1.9	8.6–16.8	
P2	Right	5.7	1.3	3.2–9.2	0.104
	Left	5.3	1.4	3.0–8.9	
DD	Right	1.0	0.2	0.6–1.8	0.274
	Left	1.0	0.3	0.4–1.9	
HL	Right	30.1	2.4	26.1–36.9	0.719
	Left	30.0	2.3	26.2–36.7	
RN-O	Right	17.7	1.1	16.1–19.3	0.824
	Left	17.8	0.9	16.6–19.4	

No statistically significant differences were documented in all parameters

*DD* direct distance between tip of olecranon and anconeus branch, *HL* humeral length, *P1* medially/centrally orientated lateral para-olecranon approach 1, *P2* laterally orientated lateral para-olecranon approach 2, *RN-O* distance between distal tip of olecranon and radial nerve, *SD* standard deviation

**Table 2:** Data analyzed per sex

Parameter	Sex	Mean (cm)	SD (cm)	Range (cm)	*p* value
P1	Male	12.7	2.0	10.6–16.8	**0.002**
	Female	11.7	1.5	8.2–13.7	
P2	Male	5.9	1.4	4.5–9.2	**≤ 0.001**
	Female	5.0	1.2	3.0–7.6	
DD	Male	1.1	0.2	0.4–1.2	**0.002**
	Female	1.0	0.3	0.7–1.9	
HL	Male	31.1	2.3	29.3–36.9	**≤ 0.001**
	Female	28.8	1.6	26.1–29.9	
RN-O	Male	18.6	0.6	17.5–19.4	**0.002**
	Female	17.1	0.6	16.1–18.3	

Statistically significant differences were documented in all parameters. Respective statistically significant *p* values are marked bold.

*DD* direct distance between tip of olecranon and anconeus branch, *HL* humeral length, *P1* medially/centrally orientated lateral para-olecranon approach 1, *P2* laterally orientated lateral para-olecranon approach 2, *RN-O* distance between distal tip of olecranon and radial nerve, *SD* standard deviation

**Table 3:** Data analyzed per humeral length

Parameter	Length	Mean (cm)	SD (cm)	Range (cm)	*p* value
P1	Long	13.0	1.6	9.1–16.8	**≤ 0.001**
	Short	11.5	1.7	8.2–14.5	
P2	Long	6.0	1.3	3.6–9.2	**≤ 0.001**
	Short	5.0	1.2	3.0–7.7	
DD	Long	1.1	0.3	0.7–1.8	**0.024**
	Short	1.0	0.4	0.4–1.9	
HL	Long	31.8	1.8	29.7–36.9	**≤ 0.001**
	Short	28.2	1.3	26.1–29.6	
RN-O	Long	18.3	0.9	17.6–19.4	**0.008**
	Short	17.2	0.7	16.1–18.7	

Statistically significant differences were documented in all parameters. Respective statistically significant *p* values are marked bold.

*DD* direct distance between tip of olecranon and anconeus branch, *HL* humeral length, *P1* medially/centrally orientated lateral para-olecranon approach 1, *P2* laterally orientated lateral para-olecranon approach 2, *RN-O* distance between distal tip of olecranon and radial nerve, *SD* standard deviation

## Discussion

The following research questions were validated: (A) a surgical guide for preserving the motor function of the anconeus branch with a corresponding nerve protection zone; and (B) a confirmation for sex and humeral length-dependent differences in both approaches. When performing P1, the anconeus branch did not come closer than 8.2 cm proximal to the olecranon in any specimen. The investigated hypothesis, stating that a broader safe zone could be reached using a standardized PA, can therefore be accepted for P1.

Maniglio et al. [21] revised the anatomy of the anconeus branch in 15 elbows, and their results are well comparable to our topographical results in a larger collective. The anconeus branch separated from the radial nerve at mean 16.4 cm from the lateral epicondyle, which is similar to our mean of 17.7 cm measured from the olecranon. Remembering our study design based on easily reproducible intraoperative anatomical landmarks, our measurements were taken starting from the olecranon. These values are well comparable due to the close anatomical relationships. Our findings also agree regarding the close relationship to the elbow joint capsule, which is probably the most vulnerable section of the nerve. Molinier et al. [22] reported a similar course of the anconeus branch and descript branches innervating parts of the triceps muscle.

Özer et al. [23] evaluated the course of the anconeus branch in 14 upper extremities. The average distance of the exit point from the radial nerve was 16.8 cm proximal to the medial humeral epicondyle. Our mean distance was similar with 17.7 cm from the olecranon to the exit point of the radial nerve. Özer et al. [23] reported that the branch coursed along the posterior humerus at an average distance of 14.2 cm medial and 4.8 cm lateral to the olecranon with regard to the described reference lines. We found additionally equivalent results with a mean intersection point of 12.3 cm (P1) and 5.5 cm (P2) from the olecranon to the anconeus branch with regard to the described approaches.

Jiménez-Díaz et al. [24] reported a high-frequency double-innervation pattern from a branch of the posterior interosseous nerve to the anconeus muscle, which was present in 70.4% of their sample. The entry point of this accessory branch was always observed distal and medial to the lateral epicondyle. This accessory branch can be disregarded in a PA, because it would be injured by lateralizing the anconeus muscle. As in our results, the main anconeus branch was present in all specimens. Here, the mobilization of the anconeus muscle in a lateral direction preserves the classical innervation. Care should be taken of the nerve at its course adjacent to the elbow joint capsule. Further, the surgeon should strictly stay near the olecranon under preservation of a small aponeurotic stripe for closure of the fascia distally. We observed a transverse direct distance to the olecranon of 1.1 cm at mean in our collective, which showed an adequate distance to the olecranon. Jiménez-Díaz et al. [24] reported a comparable horizontal distance of 1.7 cm at mean between the lateral epicondyle and the entry point into the anconeus muscle in 90° flexion of the elbow joint.

Surgeries of the elbow region may be technically demanding procedures, which demand an accurate soft tissue management. We prefer the P1—if applicable—and argue for a nerve- and muscle-preserving strategy, which is possible and supported by the reported data. In 2000 O’Driscoll et al. [25] introduced the triceps reflecting anconeus pedicle approach as an inter-nervous plane approach in an attempt to avoid denervation.

The anconeus muscle could have a significant contribution in force production and produces up to 15% of the extension torque [26]. The muscle is considered as a multifunctional muscle, which contributes in extension and stability of the elbow [27]. Molinier et al. [22] and Pereira [28] concluded that the anconeus muscle is an active lateral ligament of the elbow and constrains to the posterolateral stability due to its close relationship to the triceps brachii muscle, the lateral collateral ligament and the elbow joint capsule.

This study had several limitations. First, each specimen was used for both approaches. In this regard, it must be underlined that all the measurements were accomplished without any effect for interpretation. The randomized sequences had similar values (*p* > 0.05) and no differences regarding the increase or decrease of the safe zone in P1 and P2. Second, the measurements were taken in a highly elective anatomy environment; in a traumatic setting, potential changes have to be considered. A further limitation might be the fact that all the body donors were of European origin, and anatomical variations in different human populations must be considered. Postmortem and embalming-related effects such as denaturation and degreasing may have also influenced the results of the presented morphometry [12, 13].

Up to now, it was unclear whether there is any nerve-free zone in which a PA can be performed without risking nerve damage. To the best of our knowledge, there are no studies that address how the surgical approach affects the anconeus branch. Moreover, there exist no data on the possibility of radiological localization using preoperative magnetic resonance imaging or preoperative/intraoperative ultrasound of the anconeus branch. Both tools—especially the magnetic resonance imaging—are not well applicable in trauma cases. Thus, this study reports novelty as it was performed under a consistent, reproducible algorithm in a representative cohort of 120 specimens. An additional fortification represents the independent observer interpretation and their sufficient consensus. The observer’s agreements with different levels of experience correspondingly support the data.

## Conclusion

A nerve-free zone of 8 cm proximal to the olecranon could be validated in the P1, which enables to sparing of the anconeus branch and minimizes the risk of nerve injury during surgery. In a laterally orientated triceps-splitting technique like the P2, the anconeus branch is at risk. A reduced safe zone for female and shorter upper extremities must be taken into account.

Thus, we recommend a centrally orientated approach, which characterizes a viable nerve- and muscle-preserving strategy for a free-functional aftercare in joint preservation or replacement treatments. This might promote improved postoperative extension and represent an optimization for complex clinical management such as revision or salvage procedures.
